# The Story of the Insane from Year to Year

**Published:** 1899-04-29

**Authors:** 


### The Institutional Workshop.

### THE STORY OF THE INSANE FROM YEAR TO YEAR.

(Eleventh Series.)

### THREE COUNTIES ASYLUM AT ARLESEY.

This asylum contained 1,076 patients on January 1st, 1897, and 1,085 on December 31st. There were 230 first admissions, 50 readmissions, 106 recoveries, 12 discharged relieved, and 121 deaths. Calculated on the admissions, the recoveries work out at 34·3 per cent., which is a shade higher than their average for the last 30 years; and the deaths stand at 11·29 per cent. of the average number resident, which is rather higher than their own average, and also above the general county asylum average. Forty-five of the deaths were caused by cerebral and spinal diseases, 13 by consumption, and 6 by influenza. Of the year's admissions 42 owed their insanity to one or other of the various moral causes, 19 to intemperance in drink, 101 to hereditary influence, and 36 to previous attacks. The asylum contains about 50 patients above the number for which it was constructed, but the overcrowding will be relieved on the opening of the new asylum for Hertfordshire. The visitors record their regret at the loss the asylum sustained by the death of Dr. Evans, one of the assistant medical officers. The average cost of maintenance per head per week is 8s. 7d.

### THE MONMOUTHSHIRE ASYLUM AT ABERGAVENNY.

During the year 1897 this asylum increased the number of its patients by 37, and on the last day of the year it contained 1,012. There were 162 first admissions, 34 readmissions, 62 recoveries, 13 discharged relieved, and 74 deaths. The recoveries stand at 31·6 per cent. of the admissions, and the deaths at 7·4 of the average number resident. Thirty-six of the deaths were caused by cerebral and spinal diseases; 11 by consumption, 3 by bronchitis, and 2 by pneumonia. Of the year's admissions 18 owed their insanity to moral causes, 21 to intemperance in drink, 45 to hereditary influence, and 42 to previous attacks. Courses of lectures on nursing are given by the medical officers, and 18 of the staff entered for the examination of the Medico-Psychological Association, and 17 of them obtained the nursing certificate. Each successful candidate was granted by the committee a gratuity of £2, a silver medal, and an addition to the wages. Such liberal treatment will, we hope, induce Dr. Glendinning to institute a three years' course, so that each member of his staff may have an opportunity of being trained in all branches of nursing, and of obtaining a certificate which would prove really useful to them under any circumstances. The committee express their "high appreciation of the services of Dr. Glendinning and his able assistants, Dr. Nelis and Dr. Black."

### NORTHAMPTON COUNTY ASYLUM.

On January 1st, 1897, this asylum had 895 patients in residence, and on the last day of December the number had fallen to 891. There were 148 first admissions, 27 readmissions, 55 recoveries, 21 discharged relieved, and 86 deaths. The recoveries stand at 34·59 per cent. of the admissions, and the deaths at 9·6 per cent. on the average number resident. Of the deaths, 40 were caused by cerebral and spinal diseases, and 15 by consumption. Of the year's admissions 27 owed their insanity to various moral causes, 6 to intemperance in drink, and 24 to hereditary influence; 44 are returned as not ascertained. The post-mortem examinations only reached 38, but Mr. Greene thinks this is not an unsatisfactory proportion to the deaths, and he explains that at Berrywood "these examinations are never made except with the full approval of the relations of the deceased." We are pleased to note that the feelings of the poor relatives are respected in this asylum, and we trust these principles will soon spread to those places where a boast is made that post-mortem examinations are made in every case of death. Mr. Greene resigned the post of medical superintendent at the close of the year, and he concludes his report in the following words: "It is often said that no one ever performs an habitual action for the last time without regreting it. In presenting this report the medical superintendent feels the truth of that saying to its fullest extent, and so assured is he that the visitors appreci ate and understand his feelings that he need not enlarge on them; but after a service of nineteen and a half years of unbroken harmony, he merely expresses his thanks and his farewell in language which cannot fail to be all too inadequate to the occasion." The visitors granted Mr. Greene a handsome pension, and they add that "he will take into retirement from public work the cordial thanks and approval of the committee in which it is hoped that the whole Council will concur.' Dr. Harding was appointed medical superintendent. The average weekly cost of maintenance per head was 7s. 4½d.

### BETHLEHEM HOSPITAL.

The numbers in this hospital increased by 16 during the year, but there were only 227 in residence, so that even as regards numbers no satisfactory comparison can be made between this institution and our county asylums; and when we come to the recovery rate and death rate it is useless to compare them, as the class of patients differs considerably. We find there were 179 first admissions, 42 readmissions, 84 recoveries, and 21 deaths. Put into percentages the recoveries work out at 38 per cent. on their admissions, which is much below their average; and the deaths at 9·2 per cent. on the average number resident. Of the year's deaths 15 were caused by cerebral and spinal diseases, and 1 each by consumption, diabetes, and congestion of the lungs. Intemperance in drink figures as the exciting cause of insanity in 10 of the year's admissions, and hereditary influence as the predisposing cause in 103. Voluntary boarders are received in this hospital, and 46 of these had been under treatment during 1897, 13 of whom remained on the date of the report. A considerable amount of renovation was carried out, and at the women's side of the house several patients had to be denied admission while the repairs were in progress. We notice that Dr. Smith has put spring latches on the doors of the single-bedded rooms, but he does not tell us what he thinks of the alteration. Probably after one of the night staff finds himself hermetically sealed with a homicidal patient the old order of things will be restored. The assistant medical officers (Drs. Hyslop and Craig) give instructions on nursing subjects to the staff, and eight nurses and two attendants have passed the examination for the certificate of the Medico-Psychological Association. We venture to hope that Dr. Smith will see his way to extend these courses of lectures, and train his nurses for a three years' "Bethlem certificate."

### KENT COUNTY ASYLUM AT CANTERBURY.

There were in residence 900 patients at the beginning of the year 1897 and 944 at the end of it. There were 185 first admissions, 24 readmissions, 74 recoveries, and 75 deaths. The recoveries, calculated in the usual way, stand at 42 per cent. This is above the county asylum average and also about their own average. The deaths stand at 8 per cent. of the average number resident. Thirteen of the deaths were caused by cerebral and spinal diseases, 13 by consumption, 8 by pneumonia, 3 by bronchitis, and 3 by enteritis. Reference to Table X. shows us that of the year's admissions 27 owed their insanity to such moral causes as domestic trouble, mental anxiety, and religion; 26 to intemperance in drink; 71 to hereditary influences, and 46 to previous attacks. The asylum is practically full, but additions are being made to it. The average weekly cost of maintenance is 9s. 6d. per head.

